# Cannabis Use in Opioid Maintenance Therapy: Prevalence, Clinical Correlates and Reasons for Use

**DOI:** 10.3390/brainsci15070699

**Published:** 2025-06-29

**Authors:** Markus Backmund, Greta G. Zámbó, Susanne Schöfl, Michael Soyka

**Affiliations:** 1 Ludwig-Maximilians-University Munich, Munich, Germany; 2 P3 Clinic, Tutzing, Germany; 3 PIT—Outpatient Treatment Center, Munich, Germany

**Keywords:** cannabis, cannabis dependence, opioid use disorder, maintenance therapy, substitution, methadone, buprenorphine

## Abstract

Background and aims: Opioid maintenance therapy (OMT) is the first-line treatment for opioid use disorder (OUD), reducing opioid use and mortality while improving physical and mental health. However, concomitant substance use remains common, with cannabis being the most frequently used substance. This study assessed the prevalence and clinical correlates of cannabis use in OMT patients, as well as individual motivations. Methods: In this cross-sectional, single-center study, 128 OUD patients (96 male, 32 female) receiving OMT were assessed using standardized questionnaires: the Marijuana Smoking History Questionnaire (MSHQ), Cannabis Problems Questionnaire (CPQ) and the Severity of Dependence Scale (SDS). Cannabis users and non-users were compared regarding type (methadone vs. buprenorphine) and dosage of maintenance medication. Results: Cannabis use was reported by 41% of patients, 73% met criteria for cannabis dependence, 30% of the full sample. Of the patients, 85% reported cannabis-related legal issues. Common reasons for use included recreational motives (mood change, enhancement) and reduction in cravings for other substances. Cannabis dependence was significantly more common in patients receiving buprenorphine than methadone. Higher methadone doses were also associated with increased cannabis use. These results suggest a clinically relevant pattern. Conclusions: Cannabis use is highly prevalent and appears to be influenced by type and dosage of substitution medication. These findings highlight a complex interaction between opioid treatment and cannabis use, possibly involving behavioral coping or regulatory processes. Further longitudinal and placebo-controlled trials are needed to investigate the clinical and pharmacological interactions between cannabis and OMT, including effects on craving, withdrawal, and overall treatment outcomes.

## 1. Introduction

About 40 million people worldwide abuse opioids [1]. Prevalence rates for opioid dependence in Western countries are estimated at 0.3–0.4% [1,2]. In addition to the use of heroin or other illegal opioids, the United States is facing an opioid crisis, with many individuals dependent on prescription or pharmaceutical opioids such as fentanyl [1,2,3]. Thus, opioid use disorders (OUD) remain a major public health issue. Comorbid substance use is frequent in OUD, particularly involving cannabis or other psychotropic substances. The relationship between cannabis and opioid use is complex, with cannabis often viewed as a starter drug. Some studies suggest that cannabis users are more likely to initiate opioid use, but the overall quality of studies on this issue is limited [4].

Opioid maintenance treatment (OMT) remains the first-line treatment for OUD. Multiple lines of evidence demonstrate that OMT is very effective in reducing opioid use, criminality and mortality, while improving physical and mental health as well as social integration [5,6]. Methadone and buprenorphine are the most commonly used maintenance drugs [7]. There is some evidence that patients in buprenorphine treatment may have a lower mortality risk compared to methadone patients [6], but also a higher risk for additional substance use [7], although this issue is controversial. Individuals with opioid use disorder also often suffer from comorbid psychiatric disorders and report use of other substances [8,9].

Cannabis is the most widely used illicit drug worldwide, still being illegal in many countries [10,11]. Among general population users, relaxation and social motives are commonly cited [12]. Previous research suggests that 35–51% of individuals with OUD also use cannabis, significantly more often than in the general population [13,14]. Among non-OUD cannabis users, the rate of physiological dependence is estimated to be 10–15% [15].

With respect to cannabis use in opioid users, Budney et al. [16] reported that 66% of cannabis users in treatment were individuals with OUD. Balhara and Jain [17] reported a prevalence rate of 32%. A large Canadian study reported that 51% of patients in pharmacological treatment for OUD used cannabis in the past month [18]. Daily cannabis users had lower odds for opioid use compared with less regular or occasional cannabis users.

Some studies suggest that cannabis may have effects on opioid use as well as the course of comorbid psychiatric disorders [15,16,17,18,19,20,21,22]. Dupont and Saylor [23] reported an adverse outcome in patients with methadone maintenance who were also marijuana users. Wasserman et al. [24] also found a higher risk of lapse to heroin. In contrast, possible benefits of cannabis in OUD have also been discussed [25]. Cannabis, with its active compounds delta-9-tetrahydrocannabinol (THC) and cannabidiol (CBD), stimulates appetite and is an effective antiemetic, antispasmodic, and analgesic drug [25]. There is a close interaction between the endocannabinoid and the opioidergic system. Cannabinoid 1 (CB1) and μ-opioid receptors (MOR) are displayed in the same brain regions and there is a bidirectional relationship between MOR and CB1 [25,26,27,28]. Methadone is a full, buprenorphine a partial agonist at the MOR receptor. Cannabis may be used to alleviate acute opioid withdrawal, for pain management and as a harm reduction tool in OUD [25].

There is some preclinical evidence that CB1 receptors play a role in opioid withdrawal and cannabinoid antagonists may reduce rewarding effects of opioids, [29,30] although human evidence remains inconclusive [31]. Some studies suggest cannabis may reduce opioid use or prevent escalation [32,33], although there are clearly conflicting results from other studies [34,35,36].

Most of the existing literature on cannabis use is related to pain management rather than opioid use disorders. A large study from Lucas et al. [37] found medicinal cannabis to reduce opioid use in pain patients. Some studies have shown CBD can reduce opioid withdrawal symptoms and improve abstinence rates in cannabis users [38,39]. There are preclinical data suggesting that CBD alters opioid-related behavior, with animal models showing a decrease in the morphine-dependent reward response in the brain [40,41] and a reduction in relapse rates to morphine [41,42,43].

To date, limited clinical data are available on the effects of cannabis in individuals with OUD. A clinical pilot study by Hurd et al. [42] included 17 opioid users who reported a reduction in opioid craving after taking CBD. There are inconsistent clinical data regarding the effects of cannabinoids on the severity of opioid withdrawal symptoms [43]. In a randomized, double-blind, placebo-controlled trial with 66 patients with OUD, a significant reduction in opioid withdrawal symptoms was reported following the use of 30 mg dronabinol per day. The positive effects were primarily observed in the first few days of opioid withdrawal; treatment did not alter adherence to therapy [44]. In a further double-blind, randomized, placebo-controlled trial with 12 patients, the tolerability of dronabinol was studied during an oxycodone-based opioid withdrawal. Dronabinol doses from 20 to 40 mg induced marked side effects, primarily persistent tachycardia and anxiety [45].

Scavone et al. [46] showed high rates of cannabis use both prior to and during methadone maintenance therapy, declining significantly following dose stabilization. These results may suggest that cannabis alters the severity of opioid withdrawal symptoms. Hermann et al. [47] showed different results. Of the 89 people in OMT, 37% stated that cannabis worsened their opioid withdrawal symptoms and 15% did not perceive any effect and half of those questioned reported a reduction in severity. Nava et al. [48] did not reveal any changes in heroin cravings or withdrawal symptoms in 121 people in OMT. As stated above, the Rosic et al. [18] study found daily cannabis use in OMT patients to be associated with less opioid use compared to less frequent use.

The question if cannabis use may reduce opioid use in OMT is of significant clinical relevance. Consequently, further studies on this subject are needed [38,49].

This study aimed to investigate: (a) the prevalence of current cannabis use in OMT patients, (b) the prevalence of cannabis dependence, (c) individual motivations for cannabis use, and (d) potential differences in cannabis use between patients treated with methadone and those receiving buprenorphine.

## 2. Materials and Methods

This cross-sectional, single-center study was approved by the Ethics Committee of the Ludwig-Maximilians-University of Munich (Project Number 22-0796). The study was conducted in May 2020. All patients undergoing OMT at the outpatient addiction treatment center “Praxiszentrum im Tal” at that time were invited to participate. Inclusion criteria were a minimum age of 18 years, a current ICD-10 diagnosis of opioid dependence, ongoing OMT, and sufficient proficiency in German. Participants had to provide written informed consent. Individuals with insufficient German language skills were excluded.

Data were collected through a structured 45 min face-to-face interview using standardized instruments. All interviews were conducted by a single, trained interviewer to ensure consistency. In addition to self-report data, patient records and routine urine toxicology screens were reviewed to corroborate the reported substance use patterns.

The following validated German-language questionnaires were used: the Marijuana Smoking History Questionnaire (MSHQ), focusing on patterns and frequency of use (21 items); [50,51] the Marijuana Motives Measure (MMM), assessing motives for use (27 items); [52] a modified version of the Marijuana Ladder (ML; the Government of the Hong Kong Special Administrative Region: Narcotics Division 2012) [53,54] the Cannabis Problems Questionnaire (CPQ, 22 items); [54] and the Severity of Dependence Scale (SDS), a five-item screening tool with a scoring range of 0 to 4 [55]. Furthermore, semi-open and open-ended questions were included to explore individual motives for cannabis use in greater depth.

Cannabis dependence was classified based on SDS scores, using sex-specific cut-offs as proposed by Gossop et al. [56] with thresholds of >4 for males and >2 for females. Those reporting no cannabis use were assigned a score of 0 and considered non-dependent. The two groups—cannabis-dependent and non-dependent—were then compared with respect to their type of opioid maintenance medication and dosage.

Data were analyzed using Microsoft^®^ Excel for Mac (Version 16.16.21) and IBM^®^ SPSS^®^ Statistics (Version 25). Descriptive statistics included frequencies, means with standard deviation (SD), medians, and range values. For group comparisons, chi-square (χ^2^) four-field tests were used for categorical variables, *t*-tests for normally distributed continuous data, and Mann–Whitney U tests for non-normally distributed data. Spearman’s rank correlation was applied to assess relationships between two abnormally distributed metric variables. Results are reported with Spearman’s rho *r_s_*- and *p*-values, and the coefficient of determination (*r*^2^). A significance level of α = 0.05 was defined, and *p*-values below this threshold were considered statistically significant. Effect sizes (Cramér’s V, Cohen’s d) and 95% Confidence Intervals (CI) were calculated to assess the clinical relevance of statistically significant findings. For group comparisons of categorical variables, Cramér’s V was reported alongside chi-square statistics, with 95% confidence intervals computed using the Bonett method. For comparisons of continuous variables between two groups, Cohen’s d was calculated with 95% confidence intervals based on pooled standard deviations.

## 3. Results

In May 2020, 128 patients (96 male, 32 female) were enrolled in OMT at the “Praxiszentrum im Tal” outpatient facility. The average age was 45 years (range: 21–69, SD = 9). The mean duration of opioid use was 21 years. Among all patients, 66 (52%) were treated with methadone, 41 (32%) with buprenorphine, 4 (3%) with a buprenorphine/naloxone combination and 12 (9%) with retarded morphine. Given the small size of the morphine subgroup, separate analysis was not performed.

A total of 53 patients (41%) reported regular cannabis use, while 75 (59%) reported no use. In 95% of cases, cannabis use preceded opioid use, on average by three years. In 91% of cases, self-reported regular cannabis use was confirmed by urine drug testing. THC was also detectable in blood samples in 51% of users, which likely reflects differences in detection windows between matrices. Men and women did not differ significantly in cannabis use prevalence. The average duration of cannabis use was 24.7 years, with a mean daily consumption of 1 g (range: 0.1–5.0 g/day). The average age at initiation of use was 14 years (range 12–35 years). Of the users, 80% consumed cannabis daily, 12% once a week, and 7% once a month. Marijuana was used by 90%, hashish by 71%, and cannabis oil by 15%. Most patients (90%) smoked joints, while 20% reported using bongs and 10% used pipes. None of the patients reported using prescribed medical cannabis. Monthly spending on cannabis averaged €100 (range: €0–650).

**Figure 1:**
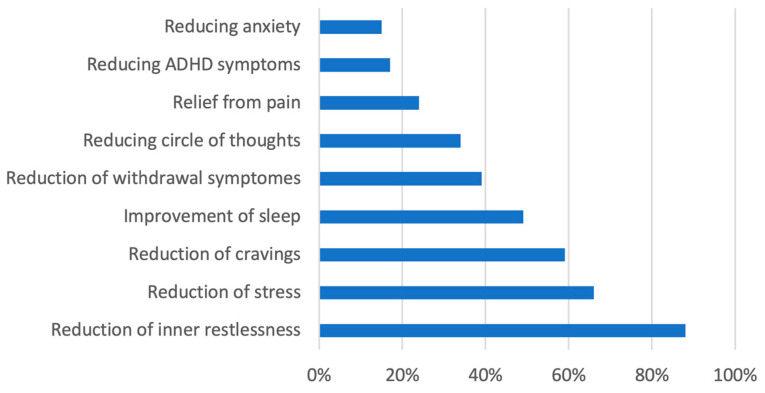
Motives for cannabis use.

Cannabis users often reported concurrent use of other substances: all were tobacco smokers, 44% used alcohol, 41% benzodiazepines, and 22% pregabalin. The most commonly endorsed reasons for cannabis use, as measured by the Marijuana Motives Measure, related to mood and affect regulation: “Because it gives me a pleasant feeling” (94%), “Because I like the feeling” (84%), and “Because it’s fun” (72%). In response to semi-structured questions, 88% cited relief from inner restlessness as a reason, while 59% mentioned the reduction in cravings for other substances (Figure 1).

**Figure 2:**
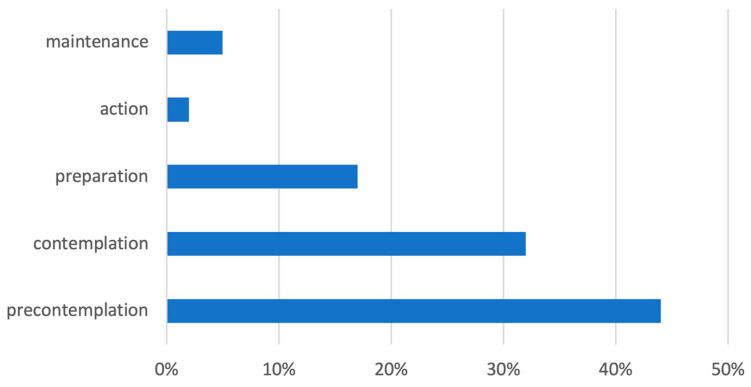
Results of the marijuana ladder.

Motivation to change cannabis use, as assessed by the Marijuana Ladder, varied across stages: 44% of patients were in the precontemplation stage (not engaged in a change in use), 32% in contemplation (planned to change their use), 17% in preparation (had already planned a change), 2% in action (stage of change), and 5% in maintenance (Figure 2).

According to the Cannabis Problems Questionnaire, 85% of users reported legal issues, such as police citations or fines related to cannabis possession; 39% expressed no intention to change their use, while 36% associated their use with depressive symptoms, and 20% reported physical complaints.

The Severity of Dependence Scale identified 73% of cannabis users as dependent (67% male, 57% female), representing 30% of the total sample. No significant differences in age, gender, or comorbidities were observed between cannabis-dependent (group 1) and non-dependent participants (group 2).

**Figure 3:**
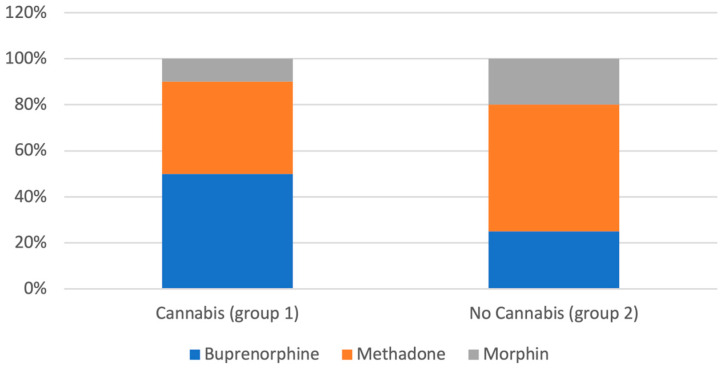
Substitution medication in cannabis-dependent patients (Group 1) and non-dependent individuals (Group 2).

Of all patients, 55% received methadone, 36% buprenorphine and 9% slow-release morphine. Among cannabis-dependent patients (group 1), buprenorphine was more commonly prescribed (50%) compared to methadone (40%) and morphine (10%), a distribution that differed significantly from the overall sample χ^2^(1) = 4.24, *p* = 0.039, Cramér’s V = 0.18, 95% CI [0.01, 0.34]), indicating a small to moderate effect size (Figure 3).

Moreover, methadone dosage differed significantly between the two groups: dependent cannabis users received higher doses (mean 119.2 mg) than non-dependent patients (mean 85.2 mg; *p* = 0.031, Cohen’s d = 0.61, 95% CI [0.06, 1.16], indicating a moderate effect size). There was a positive correlation between methadone dose and daily cannabis use (Spearman’s ρ = 0.34, *p* = 0.01). No significant difference in buprenorphine dosage was observed.

## 4. Discussion

This study is in line with previous research indicating a high prevalence of cannabis use in OUD and OMT [14,15,16,17,18,20]. Cannabis use was frequent in this OMT sample with 41% of the 128 patients reporting cannabis use. Other studies also indicate a high prevalence of about 35–50% of cannabis use in patients with opioid use disorder [13,14]. While cannabis dependence in nonopioid dependent cannabis users is the exception, with prevalence rates not exceeding 10–15% [15] in this sample a rate of 73% for cannabis dependence was found among cannabis users. In total, the prevalence of cannabis dependence in the group of patients in OMT was, therefore, 30%. Patients in this sample on average had a long history of cannabis use of over 20 years.

The interplay between cannabis and opioid use appears to be complex. Whether cannabis dependence per se is a risk factor for OUD or whether OUD facilitates the progression from cannabis use to dependence is a controversial issue [4].

With respect to the general population with a higher prevalence of substance and cannabis use in males and younger individuals between 15 and 24 years of age [8,9] (with respect to gender and age distribution), no significant differences could be demonstrated in major sociodemographic variables for cannabis use/dependence in this OMT population. Important data on this issue come from a recent publication of a 20 year follow-up of the Australian ATOS study on 615 people with heroin dependence [57]. An increase in cannabis use 24 months after baseline was significantly associated with an increase in heroin use after 36 months and increase in heroin use at 3 months and 24 months was significantly associated with a decrease in cannabis use at 12 months and at 35 months. All other associations tested were negative. Overall, the authors concluded that there was insufficient evidence to suggest a unidirectional or bidirectional relationship between the use of these substances.

Given the high prevalence of psychiatric and somatic comorbidities, cannabis may have beneficial effects on certain symptoms [1,13,15]. Recently, a comprehensive meta-analysis (10 studies, 8367 participants) on the impact of cannabis on non-medical opioid use among individuals in OMT was published [58]. Costa et al. [58] did not find a significant association between cannabis and opioid use among patients receiving pharmacotherapies for OUD suggesting an individual approach for cannabis use. To date, there are few data on individual reasons for cannabis use in patients in OMT. In this sample, most patients reported cannabis use for recreational reasons but some also to alleviate psychopathological symptoms such as inner restlessness. At 49–51%, social reasons and motives were significantly more likely to be reported as reasons for use than for the general population with 4–26%. The fact that cannabis was frequently used by patients in OMT patients to alleviate psychopathological symptoms, such as negative/depressive mood or anxiety (40%) and inner restlessness (88%), may be viewed in light of the self-medicating hypothesis in substance use disorders [59] linking unpleasant affective states such as depression or other psychopathological symptoms, unwanted side effects or inadequate dosing of medication with substance use. In addition, craving reduction (59%) was frequently reported as a reason for cannabis use by 59%.

There is very preliminary evidence for cannabis as a possible pharmacological adjunct in OUD [25]. The finding that 39% of cannabis users were found to take cannabis to suppress opioid withdrawal symptoms and 59% to suppress cravings for other substances is in part supported by previous studies [43,44,45,46] and may indicate an insufficient suppression of opioid withdrawal symptoms and craving with the existing opioid medication in this sample.

Some authors have hypothesized that cannabis might mitigate some of the negative effects of opioids and counteract inadequate methadone dosing [60] and alleviate opioid withdrawal [61]. Recent research has indicated that lower methadone dose is associated with poor retention rates regardless of concurrent high-frequency cannabis use [62].

Whether cannabis use is related to dose of the OMT medication is an open question. Rosic et al. [18] failed to find an association of past month cannabis use with more or less opioid use during treatment. Some studies found a negative impact on outcome in OMT [24], others not [15,63]. At least in this study, patients at higher doses of methadone were more likely to use cannabis than patients at lower dosages. This may indicate that cannabis use is not primarily related to OMT dosage in patients. Further randomized, placebo-controlled and longitudinal trials with medicinal cannabis are necessary to further elucidate the effects of cannabis on opioid use disorder. Unfortunately, there still are significant legal barriers on this issue [64].

Finally, the role of different opioid medications for cannabis use in OMT must be addressed. This study, for the first time, shows that the cannabis use pattern may differ between patients treated with buprenorphine with those who are treated with methadone. The rate of cannabis dependence in the buprenorphine group was significantly higher compared to the methadone group (*p* < 0.05). Notably, the average buprenorphine dose was the same for non-cannabis-dependent and cannabis dependent individuals, different to findings by Balhara and Jain [17]. There is little research on this issue. There are anecdotal reports about adverse reaction of cannabis use and buprenorphine [65]. In contrast, cannabis dependent patients receiving methadone were treated with 119 mg methadone on average, a significantly higher dose compared to 85 mg among the patients who were not cannabis dependent. A possible explanation could be that patients with a more severe level of dependence and intense craving require higher dosages of methadone and also are more prone to additional cannabis use. In over 90% of cases, cannabis use precedes opioid use [66].

In the light of ongoing changes in cannabis legalization in many countries, the possible consequences of an increasing cannabis use in OMT may be discussed. There were initial findings indicating a reduction in opioid-related deaths [67,68] but more recent data linked an increasing cannabis use to more negative outcomes [69,70]. Longitudinal studies do not indicate that reductions in heroin use are associated with increase in cannabis or other substance use [71].

There are several limitations with this study. Due to the cross-sectional design, causal inferences regarding the relationship between cannabis use and OMT parameters are limited. Data on psychiatric comorbidities were extracted from clinical records and approximated via the number of prescribed psychotropic medications. While this provides an indirect estimate, the absence of standardized psychopathological assessments restricts conclusions about potential psychological confounders. Furthermore, the sample showed a marked predominance of male participants. However, this reflects the typical demographic pattern in opioid maintenance treatment, where men are significantly overrepresented. All eligible patients receiving OMT at the treatment center during the recruitment period were included, and no intentional selection bias was introduced. Lastly, the findings are based on data from a single treatment center, which may limit generalizability. The chronological relationship between opioid use and cannabis use in this sample of predominantly long-term users could not be assessed retrospectively. Longitudinal studies are necessary to explore the interrelationship between opioid maintenance therapy, choice of medication and dose of cannabis, and whether there is a progression or regression of cannabis use over time. Second, the sample size, especially with respect to subtypes of patients or groups, was limited and does not permit generalizability of patients. Third, a limited number of assessments were included in this study to ensure practicability. Reasons for use may also be explored in more detail in longitudinal studies with more detailed psychometric assessments. Larger studies are necessary to further study reasons and motives for use and cannabis effects on mood or well-being.

## 5. Conclusions

This study highlights the clinical correlates and individual motives associated with cannabis use in OMT patients. Cannabis use was significantly more common among patients receiving buprenorphine, and higher methadone doses were associated with increased cannabis use. These findings underline the clinical relevance of cannabis use in OMT and the need for further research. Future longitudinal and placebo-controlled trials should examine pharmacological interactions (e.g., plasma levels, CBD/THC effects), as well as potential benefits regarding craving and withdrawal. Preliminary evidence [42,72,73] suggest that cannabis may alleviate opioid cravings in some patients. While motives for use vary, managing cravings and drug-related side effects appear to be relevant factors.

## Data Availability

The data presented in this study are available on request from the corresponding author due to privacy.

## Abbreviations

The following abbreviations are used in this manuscript:

CBD	Cannabidiol
CB1	Cannabinoid Receptor Type 1
CI	Confidence Intervals
CPQ	Cannabis Problems Questionnaire
ICD-10	International Classification of Diseases, 10th Revision
MMM	Marijuana Motives Measure
MOR	μ-opioid receptor (Mu-Opioid Receptor)
MSHQ	Marijuana Smoking History Questionnaire
ML	Marijuana Ladder
OMT	Opioid Maintenance Therapy
OUD	Opioid Use Disorder
SDS	Severity of Dependence Scale
SPSS	Statistical Package for the Social Sciences
TCH	Delta-9-Tetrahydrocannabinol
